# Sigma metrics for assessing the analytical quality of clinical chemistry assays: a comparison of two approaches: Electronic supplementary material available online for this article.

**DOI:** 10.11613/BM.2018.020708

**Published:** 2018-06-15

**Authors:** Xiuzhi Guo, Tianjiao Zhang, Xuehui Gao, Pengchang Li, Tingting You, Qiong Wu, Jie Wu, Fang Zhao, Liangyu Xia, Ermu Xu, Ling Qiu, Xinqi Cheng

**Affiliations:** 1 Department of Laboratory Medicine, Peking Union Medical College Hospital, Chinese Academic Medical Science and Peking Union Medical College, Beijing, P.R. China; 2 National Center for Clinical Laboratories, Beijing Hospital, National Center of Gerontology, Beijing, P.R. China; 3 Clinical Laboratory, Affiliated Hospital of Chifeng University, Inner Mongolia, P.R. China

**Keywords:** Six Sigma, proficiency testing, internal quality control, allowable total error

## Abstract

**Introduction:**

Two approaches were compared for the calculation of coefficient of variation (CV) and bias, and their effect on sigma calculation, when different allowable total error (TEa) values were used to determine the optimal method for Six Sigma quality management in the clinical laboratory.

**Materials and methods:**

Sigma metrics for routine clinical chemistry tests using three systems (Beckman AU5800, Roche C8000, Siemens Dimension) were determined in June 2017 in the laboratory of Peking Union Medical College Hospital. Imprecision (CV%) and bias (bias%) were calculated for ten routine clinical chemistry tests using a proficiency testing (PT)- or an internal quality control (IQC)-based approach. Allowable total error from the Clinical Laboratory Improvement Amendments of 1988 and the Chinese Ministry of Health Clinical Laboratory Center Industry Standard (WS/T403-2012) were used with the formula: Sigma = (TEa − bias) / CV to calculate the Sigma metrics (σ_CLIA_, σ_WS/T_) for each assay for comparative analysis.

**Results:**

For the PT-based approach, eight assays on the Beckman AU5800 system, seven assays on the Roche C8000 system and six assays on the Siemens Dimension system showed σ_CLIA_ > 3. For the IQC-based approach, ten, nine and seven assays, respectively, showed σ_CLIA_ > 3. Some differences in σ were therefore observed between the two calculation methods and the different TEa values.

**Conclusions:**

Both methods of calculating σ can be used for Six Sigma quality management. In practice, laboratories should evaluate Sigma multiple times when optimizing a quality control schedule.

## Introduction

Clinical laboratory testing results are important for ensuring patient safety. Approximately two-thirds of important clinical decisions on patient management are based on laboratory test results (*1*). Therefore, continuous improvement and the minimizing of errors in testing are the major goals of every clinical laboratory. Six Sigma (6σ) quality management is a data-based, customer-centered, advanced quality management model that has been recently developed and is used globally. Following its introduction in clinical laboratories, 6σ quality management has become a major research focus (*2*). Sigma (σ) metrics evaluate process capability. The clinical application of 6σ quality management involves the combined use of quality requirements and laboratory performance to quantitatively evaluate whether a laboratory meets clinical testing standards. This evaluation is typically based on the expected defect rate. The ultimate goal of 6σ quality management is to implement laboratory risk management and thus ensure patient safety.

Several studies on the application of 6σ management in laboratory testing have been reported, including studies on theoretical methods and their significance, the evaluation of performance of different assays, and the optimization of quality control (QC) schedules based on performance evaluation (*3*-*5*). Sigma metrics quantitatively estimates quality based on the traditional parameters used in the clinical laboratory: allowable total error (TEa), bias and imprecision. Imprecision is usually expressed as standard deviation (SD) or coefficient of variation (CV). However, the quality requirements of various sources may be associated with differences within the same assay, which can affect the parameter selection. Furthermore, different approaches to bias and CV calculation may influence the final σ calculation. Laboratories should thus be aware of such sources of variation when σ management is applied, as they may modulate the value of σ and the accuracy of σ measurement. For example, previous studies have reported bias in external quality assessment (EQA) survey reports (*e.g.*, College of American Pathologists or the Randox International Quality Assessment Scheme), whereas CV is derived from the cumulative coefficient of variation of internal quality control (IQC) materials (*6*-*9*). However, a strong correlation has been established between CV and the concentration of the test substance. If the concentration of IQC materials differs significantly from that of proficiency testing (PT) samples used in bias calculation, this method is inadequate for calculating σ. Bias may also be derived from reagent package inserts (*10*). Given that the cumulative average value of QC material may change over time, it is inappropriate to use the target value from manufacturers’ QC material when calculating bias. In fact, with the continuous development of information technology, vendors can statistically analyse IQC data from the majority of laboratories to determine a more appropriate “group mean”. This approach can facilitate inter-laboratory quality management based on IQC, which has become popular among clinical laboratories. Using this group mean to calculate for bias has been shown to be a convenient and reliable method.

Therefore, in the present study, we aimed to compare two approaches to the calculation of CV and bias and the effect on σ calculation at different TEa. The two methods used to calculate the σ value for 10 routine biochemical assays on three different analysers were a PT-based approach, where materials for routine clinical chemistry from the China National Center for Clinical Laboratories (NCCL) were evaluated for imprecision (CV%) in each assay and bias (bias%) was calculated by comparison with the group mean for each PT sample in the NCCL report; and an IQC-based approach, where IQC results were used to calculate the CV% of each assay and bias% was calculated by comparison with the global group mean. Both methods thus harmonized the source of bias and CV derived from the same sample, based on which variations in σ were compared despite the different sources of parameters. This method of evaluating σ has not previously been reported.

## Materials and methods

### Materials

Ten assays were evaluated using the manufacturer/analyser combinations routinely used in Peking Union Medical College Hospital (Table 1). All reagents and calibrators for the three analysers were obtained from the original manufacturer except for creatinine (CREA; Maccura Biotechnology Co., Ltd., Chengdu, China) and bilirubin, total (BT; Wako Pure Chemical Industries, Ltd., Osaka, Japan) on the Beckman AU5800 system (Beckman Coulter, Inc., Brea, USA).

**Table 1:** Reagents, equipment and number of laboratories included in each instrument group for NCCL proficiency testing, and number of laboratories included in the Bio-Rad global report for the same instrument/method

**Analyser**	**Number of laboratories in NCCL proficiency testing/Bio-Rad global report**									
**Alb**	**ALT**	**BT**	**Glc**	**CREA**	**Urea**	**K**	**Na**	**Cl**	**Ca**
Beckman AU5800*	207/37	221/148	220/60^§^	208/48	138/22^║^	210/22	371/46	359/46	364/44	202/39
**Roche C8000^†^**	419/26	410/16	369/26	392/32	384/22	399/22	401/36	388/36	387/35	395/25
**Siemens Dimension^‡^**	7/60	6/16	6/24	8/68	7/37	8/43	8/57	8/56	8/54	7/62
*Beckman, Beckman Coulter, Inc., Brea, CA. ^†^Roche, Hoffmann-La Roche Ltd., Basel, Switzerland. ^‡^Siemens, Siemens Healthcare Diagnostics, Inc., DE, US. ^§^Wako, Wako Pure Chemical Industries, Ltd., Osaka, Japan. ^║^Maccura, Maccura Biotechnology Co., Ltd., Chengdu, China. Alb - albumin. ALT - alanine aminotransferase. BT - bilirubin, total. Glc - glucose. CREA - creatinine. K - potassium. Na - sodium. Cl - chlorides. Ca - calcium. NCCL - China National Center for Clinical Laboratories, Peking, China.										

### Methods

*Sample preparation.* PT materials (five lots: 201721–201725) for routine clinical chemistry were provided by NCCL (Peking, China). Samples were prepared using an analytical balance with an accuracy of 0.001 g. Powdered samples were dissolved in 3 mL deionized water, capped, maintained at room temperature for 30 min and gently mixed until completely dissolved. Samples were protected from light and stored between 2 °C and 8 °C until use within 7 days.

*Imprecision evaluation.* During the PT period (June 5 – 9, 2017), the Clinical Laboratory Standards Institute (CLSI) EP15A3 protocol was followed, with the same sample tested five times daily for albumin (Alb), alanine aminotransferase (ALT), potassium (K), sodium (Na), chloride (Cl), calcium (Ca), total bilirubin (BT), glucose (Glc), creatinine (CREA, enzymatic), and urea (Urea) on each analyser for five consecutive days (*11*). The mean, SD (within laboratory) and CV (within laboratory) for each test item was calculated.

*Bias calculation.* Based on NCCL routine clinical chemistry requirements, the mean value of the instrument group (excluding data more than two standard deviations away from the mean) was used to verify the target value of each assay. Our mean (N = 25) for each assay from the different analysers was calculated as described above. Bias% was determined as (our mean − mean of all laboratories using the same instrument and method) / (mean of all laboratories using the same instrument and method) x 100.

Bio-Rad (Bio-Rad Laboratories, Inc., Hercules, CA, US) Liquid assay multiqual QC materials (694/696; lot numbers 45751, 45753) were used daily to monitor internal testing quality. All assays participated in the Bio-Rad global report. Data were collected from internal QC at Peking Union Medical College Hospital from January 1, 2017 to June 30, 2017. Monthly (June 2017) mean, SD and CV were calculated. Bias was calculated based on target value, which was averaged from the Bio-Rad global report for the same assay performed with the same instrument/method.

*Sigma calculation and data analysis*. According to quality requirements, the formulas σ = (TEa − |Bias%|) / CV (for percentage) and σ = (TEa − |Bias|) / SD (for concentration value) were used to calculate σ metrics for each assay. The allowable total error of each assay was based on the American Clinical Laboratory Improvement Amendments of 1988 (CLIA ‘88) and People’s Republic of China Health Industry Standard (WS/T403-2012), designated as TEa_CLIA_ and TEa_WS/T_, respectively (*12*, *13*). The specific requirements of the 10 assays are listed in Table 2. Excel 2010 software (Microsoft Corporation, Redmond, Washington State, US) was used for data analysis and graphing.

**Table 2:** Allowable total error derived from the US Clinical Laboratory Improvement Amendments 1988 (CLIA ‘88) and the People’s Republic of China Health Industry Standard (WS/T403-2012)

**Item**	**TEa_CLIA_**	**TEa_WS/T_**
**bias% (absolute value)**	**bias%**
**Alb**	10	6
**ALT**	20	16
**BT**	20 (6.84)	15
**Glc**	10 (0.33)	7
**CREA**	15 (26.5)	12
**Urea**	9 (1)	8
**K**	(0.5)	6
**Na**	(*4*)	4
**Cl**	5	4
**Ca**	(0.25)	5
TEa_CLIA_ - allowable total error derived from US Clinical Laboratory Improvement Amendments 1988 (CLIA ‘88). TEa_WS/T_ - allowable total error derived from the People’s Republic of China Health Industry Standard (WS/T403-2012). Alb - albumin, g/L. ALT - alanine aminotransferase, U/L 37 °C. BT - bilirubin, total, µmol/L. Glc - glucose, mmol/L. CREA - creatinine, µmol/L. Urea, mmol/L. K - potassium, mmol/L. Na - sodium, mmol/L. Cl - chlorides, mmol/L. Ca - calcium, mmol/L.		

## Results

### PT Sigma metrics

The σ values calculated using two TEa sources (σ_WS/T_ and σ_CLIA_) for the three analysers are shown in Table 3. The TEa_CLIA_ used absolute bias in the K, Na and Ca assays, whereas TEa_WS/T_ used percentage bias in all other assays and was more stringent than TEa_CLIA_. We showed that σ_WS/T_ < σ_CLIA_ for all assays except Na. For σ_WS/T_, only the Siemens Dimension analyser achieved 6σ for BT testing in all five lots. The 3σ level was achieved for ALT, CREA and K on all three analysers; BT, Glc and Na on the Roche C8000 analyser; and Cl and Ca on the Beckman AU5800 analyser.

**Table 3:** Sigma metrics for the proficiency testing (PT)-based approach

**Item**	**Sample number**	**Mean**			**CV (%)**			**Bias (%)**			**σ_CLIA_**			**σ_WS/T_**		
**Beckman** **AU5800**	**Roche** **C8000**	**Siemens** **Dimension**	**Beckman** **AU5800**	**Roche** **C8000**	**Siemens** **Dimension**	**Beckman** **AU5800**	**Roche** **C8000**	**Siemens** **Dimension**	**Beckman** **AU5800**	**Roche** **C8000**	**Siemens** **Dimension**	**Beckman** **AU5800**	**Roche** **C8000**	**Siemens** **Dimension**
**Alb**	201721	46	49	51	1.0	1.4	0.8	- 1.2	- 1.0	5.8	8.4	6.6	5.2	4.6	3.7	0.3^†^
	201722	24	26	26	1.2	3.0	0.8	- 2.2	0.5	2.6	6.4	3.2	9.5	3.1	1.9^†^	4.4
	201723	32	35	35	1.0	2.1	1.3	- 1.8	- 0.4	3.6	8.5	4.5	4.8	4.4	2.6^†^	1.8^†^
	201724	46	49	52	2.3	1.6	1.1	- 1.1	- 1.5	7.2	3.8	5.5	2.6^†^	2.1^†^	2.9^†^	- 1.1^†^
	201725	37	40	41	1.3	2.1	0.8	- 2.9	- 2.2	2.9	5.5	3.8	9.4	2.4^†^	1.9^†^	4.1
**ALT**	201721	160	153	164	0.6	1.0	0.9	- 2.8	- 2.3	- 1.3	27.7	18.3	21.5	21.3	14.2	16.9
	201722	31	29	32	3.0	4.0	4.6	- 2.8	- 1.1	0.7	5.8	4.7	4.2	4.5	3.7	3.3
	201723	77	74	81	0.8	2.7	2.4	- 3.4	- 3.3	0.1	19.8	6.2	8.2	15	4.7	6.6
	201724	144	137	149	0.8	1.3	2.0	- 2.5	- 2.6	0.5	21.1	13.1	9.6	16.2	10.1	7.7
	201725	100	97	105	1.2	1.7	1.8	- 4.7	- 3.4	- 2.6	13	10	9.7	9.6	7.6	7.5
**BT**	201721	128.1	121.1	129.4	1.1	0.8	0.7	- 6.3	- 4.0	- 6.8	13	19.6	18.6	8.3	13.5	11.5
	201722	12.5	11.3	13.8	1.3	1.8	2.2	- 7.1	- 8.5	1.7	36.8*	27.6*	22.0*	6.3	3.6	6.1
	201723	25.4	23.5	27.8	0.9	1.4	1.3	- 14.6	- 7.4	0.1	11.3	15	18.9	0.4^†^	5.5	11.7
	201724	85.9	82.0	89.1	1.0	1.9	0.6	- 4.2	- 4.9	- 5.9	16	7.8	22.7	11	5.2	14.7
	201725	38.16	35.9	41.0	1.3	1.2	1.3	- 12.9	- 7.8	- 4.0	5.6	10.4	12.3	1.7^†^	6.2	8.4
**Item**	**Sample number**	**Mean**			**CV (%)**			**Bias (%)**			**σ_CLIA_**			**σ_WS/T_**		
		**Beckman** **AU5800**	**Roche** **C8000**	**Siemens** **Dimension**	**Beckman** **AU5800**	**Roche** **C8000**	**Siemens** **Dimension**	**Beckman** **AU5800**	**Roche** **C8000**	**Siemens** **Dimension**	**Beckman** **AU5800**	**Roche** **C8000**	**Siemens** **Dimension**	**Beckman** **AU5800**	**Roche** **C8000**	**Siemens** **Dimension**
**Glc**	201721	13.4	13.3	14.2	1.2	1.4	1.6	- 2.7	- 2.1	3.5	6.1	5.6	4.1	3.6	3.5	2.2^†^
	201722	3.8	3.7	4.0	1.1	1.1	2.2	- 3.0	- 2.9	5.5	6.6	6.7	2^†^	3.8	3.9	0.7^†^
	201723	6.6	6.6	7.1	1.4	1.1	2.4	- 3.1	- 2.8	4.5	5	6.9	2.3^†^	2.8^†^	4	1.1^†^
	201724	10.8	10.8	11.5	1.3	1.2	1.8	- 2.8	- 2.6	4.3	5.4	6.2	3.2	3.2	3.7	1.5^†^
	201725	7.3	7.2	7.8	1.4	0.9	1.4	- 4.5	- 4.5	2.4	3.8	6.5	5.3	1.7^†^	3	3.2
**CREA**	201721	466	464	452	0.8	1.2	0.7	- 0.2	2.1	0.4	19.4	11	21	15.5	8.4	16.7
	201722	85	88	82	0.8	1.2	2.1	9.6	5.4	0.8	28.8*	20.2*	14.9*	3.1	5.3	5.3
	201723	223	225	218	0.6	1.2	2.9	2.9	1.8	1.5	19.3	11.3	4.7	14.5	8.7	3.7
	201724	469	467	455	0.8	1.1	1.2	- 0.7	1.4	- 0.7	17.4	12.2	11.9	13.7	9.5	9.4
	201725	289	289	284	0.7	1.0	1.0	- 0.2	- 0.2	- 0.1	21.2	14.8	14.9	16.9	11.8	11.9
**Urea**	201721	17.41	17.58	18.17	2.0	3.6	1.3	- 3.9	- 2.6	0.2	2.6^†^	1.8^†^	7.1	2.1^†^	1.5^†^	6.3
	201722	4.05	4.21	4.25	2.0	3.1	3.0	- 7.0	- 1.8	- 1.0	8.8*	7.1*	7.7*	0.5^†^	2^†^	2.4^†^
	201723	9.31	9.49	9.77	2.2	3.0	1.4	- 5.1	- 2.4	- 0.4	2.5*^†^	2.6*^†^	6.9*	1.3^†^	1.8^†^	5.5
	201724	15.39	15.62	16.12	1.7	3.6	1.8	- 4.0	- 2.5	- 0.6	3	1.8^†^	4.8	2.4^†^	1.5^†^	4.3
	201725	11.11	11.28	11.76	2.4	3.3	1.9	- 6.1	- 4.2	- 0.9	1.2^†^	1.5^†^	4.2	0.8^†^	1.2^†^	3.7
**K**	201721	6.3	6.4	6.3	1.2	0.6	0.8	0.6	0.6	0.4	6.4*	12.8*	9.2*	4.7	9.6	6.9
	201722	2.7	2.8	2.7	1.0	1.2	0.9	- 2.3	- 0.5	- 2.3	16.6*	14.7*	17.3*	3.8	4.7	4
	201723	4.3	4.4	4.3	0.5	0.6	0.8	0.4	0.1	0.5	21.8*	20.0*	14.4*	10.9	10.5	7.2
	201724	6.0	6.0	6.0	0.5	0.5	0.9	1.0	0.7	0.6	14.5*	17.0*	8.6*	9.8	12	5.9
	201725	4.7	4.8	4.7	0.7	0.3	1.1	- 1.0	- 1.1	- 1.2	14.1*	31.1*	8.7*	7.3	16.4	4.4
**Na**	201721	157	159	155	0.8	0.6	0.7	0.1	0.4	- 1.4	3.2*	3.6*	1.8*^†^	5.1	6.1	3.9
	201722	108	111	112	0.8	0.9	0.8	- 2.5	0.3	1.0	1.4*^†^	3.6*	3.3*	1.8^†^	4	3.9
	201723	128	130	129	0.4	0.8	0.8	- 0.8	0.5	0.3	5.2*	3.4*	3.5*	7.1	4.5	4.6
	201724	153	154	153	0.5	0.5	1.1	0.3	2.2	0.5	5.0*	0.9*^†^	2.0*^†^	8.1	3.8	3.3
	201725	133	135	135	0.5	0.5	1.2	- 2.1	- 0.7	- 1.0	1.5^†^	5.1	1.7^†^	3.5	7.4	2.7^†^
**Cl**	201721	108	107	105	0.8	0.7	0.9	0.7	1.7	- 2.6	5.3	5	2.7^†^	4.1	3.5	1.6^†^
	201722	76	72	72	0.6	0.8	1.2	0.9	3.9	- 1.4	7.2	1.4^†^	3	5.5	0.1^†^	2.1^†^
	201723	89	86	85	0.4	0.6	0.9	0.7	2.9	- 2.0	10	3.7	3.3	7.7	2^†^	2.2^†^
	201724	108	106	105	0.6	0.5	0.6	0.8	2.1	- 0.9	6.9	5.6	6.6	5.3	3.7	5
	201725	96	94	93	0.5	0.4	0.7	- 1.1	1.4	- 2.8	7.7	9.7	3	5.7	7	1.6
**Ca**	201721	3.14	3.21	3.03	1.2	1.0	1.9	1.2	3.5	- 1.0	5.6*	4.4*	3.8*	3.1	1.5^†^	2.1^†^
	201722	1.84	1.92	1.80	1.2	0.8	1.7	1.2	4.4	- 1.3	10.5*	10.9*	7.4*	3.3	0.8^†^	2.2^†^
	201723	2.73	2.82	2.67	0.8	0.8	1.3	1.1	3.8	- 1.5	10.4*	6.5*	5.9*	5	1.5^†^	2.7^†^
	201724	3.02	3.12	2.92	0.8	0.7	1.2	0.8	4.0	- 2.0	9.1*	6.0*	5.3*	5.1	1.4^†^	2.5^†^
	201725	2.72	2.81	2.65	0.6	0.7	0.9	0.0	2.1	- 3.4	14.7*	9.9*	6.5*	8.8	4.2	1.7^†^
*TEa calculated by using absolute bias to calculate σ; not marked indicates using percentage bias to calculate σ. ^†^ Sigma values < 3. TEa_CLIA_ - allowable total error derived from US Clinical Laboratory Improvement Amendments 1988 (CLIA ‘88). TEa_WS/T_ - allowable total error derived from the People’s Republic of China Health Industry Standard (WS/T403-2012). Alb - albumin, g/L. ALT - alanine aminotransferase, U/L 37 °C. BT - bilirubin, total, µmol/L. Glc - glucose, mmol/L. CREA - creatinine, µmol/L. Urea, mmol/L. K - potassium, mmol/L. Na - sodium, mmol/L. Cl - chlorides, mmol/L. Ca - calcium, mmol/L.																

The σ_CLIA_ calculation based on TEa_CLIA_ showed that eight assays (all except Urea and Na) achieved σ > 3 on the Beckman AU5800 analyser. Among the eight, CREA and K reached 6σ levels in the five lot numbers during proficiency testing; four assays (ALT, BT, Cl and Ca) achieved 5σ. For the Roche C8000 analyser, all assays except Urea, Na and Cl achieved 3σ, whereas BT, Glc, CREA and K reached 6σ. For the Siemens Dimension analyser, BT and K reached 6σ; ALT, CREA and Urea reached 4σ; and Alb, Glc, Na (201721, 201724, 201725) and Cl (201721) were < 3σ at certain concentrations.

Significant differences in σ_CLIA_ values were observed using the same assay at the same concentration but for different analysers. More intuitive σ_CLIA_ levels for all assays at different concentrations and with different analysers are shown in Supplementary Figure 1.

### IQC Sigma metrics

The σ metrics for the ten assays calculated from internal QC data (June 2017) are shown in Table 4. Similar to the PT results, σ_WS/T_ was < σ_CLIA_ in 9 out of 10 assays (all except Na), with ALT, BT and Glc reaching 6σ; Na reaching 4σ; and K and Ca reaching 3σ on the Beckman AU5800 analyser. On the Roche C8000 analyser, Glc, CREA, K and Na reached the 6σ level; ALT, BT and Urea reached 4σ; and Ca reached 3σ. For the Siemens Dimension analyser, CREA reached 6σ, Alb reached 5σ, and Glc and K reached 3σ.

**Table 4:** Sigma metrics for the IQC-based approach using one-month IQC data

**Item**	**Control level**	**Mean**			**CV (%)**			**Bias (%)**			**σ_CLIA_**			**σ_WS/T_**		
**Beckman** **AU5800**	**Roche** **C8000**	**Siemens** **Dimension**	**Beckman** **AU5800**	**Roche** **C8000**	**Siemens** **Dimension**	**Beckman** **AU5800**	**Roche** **C8000**	**Siemens** **Dimension**	**Beckman** **AU5800**	**Roche** **C8000**	**Siemens** **Dimension**	**Beckman** **AU5800**	**Roche** **C8000**	**Siemens** **Dimension**
**Alb**	L	24	26	24	2.0	2.7	1.0	- 2.7	- 1.1	- 0.2	3.7	3.3	9.8	1.7^†^	1.8^†^	5.8
	H	39	42	40	1.9	1.4	1.0	- 2.3	- 2.3	0.6	4.1	5.5	9.4	2^†^	2.6^†^	5.4
**ALT**	L	31	29	37	1.7	3.1	2.7	0.8	3.4	11.5	10.8	5.4	3.1	8.5	4.1	1.6^†^
	H	184	173	200	0.9	1.1	1.4	0.7	1.5	8.2	17.2	16.9	8.6	12.8	13.2	5.7
**BT**	L	10.1	8.8	11.0	1.6	2.5	2.8	1.5	- 4.4	13.0	41.8*	29.3*	18.0*	8.5	4.2	0.7^†^
	H	115.4	108.2	116.6	1.3	0.9	0.7	0.6	- 2.6	- 1.2	15	19.3	26.9	11.1	13.7	19.7
**Glc**	L	3.3	3.3	3.5	1.2	1.2	2.4	- 1.2	- 2.2	1.7	4.8	4	2.2^†^	7.3	6.5	3.5
	H	20.3	20.1	20.8	1.1	1.1	1.2	- 0.5	- 1.9	0.8	5.9	4.6	5.1	8.6	7.4	7.6
**CREA**	L	78	81	75	3.3	1.7	1.4	5.3	1.3	0.6	8.5*	18.1*	24.8*	1.8^†^	6.3	8.1
	H	848	821	809	0.9	1.5	0.8	2.0	- 0.5	- 0.2	13.9	9.7	18.5	10.6	7.7	14.8
**Urea**	L	5.05	4.9	5.24	3.1	1.7	2.7	- 4.8	0.1	- 0.3	4.7*	10.1*	7.1*	1^†^	4.7	2.9^†^
	H	27.15	26.3	28.17	2.1	2.1	1.8	- 0.8	- 0.1	- 0.3	3.9	4.3	4.8	3.4	3.8	4.3
**K**	L	2.6	2.6	2.4	1.2	0.8	1.0	0.7	- 0.8	- 2.2	16.1*	24.0*	18.6*	4.4	6.5	3.8
	H	8.0	8.1	7.9	1.5	0.6	0.7	1.5	- 0.1	- 1.5	3.3*	10.8*	6.6*	3	9.9	6.5
**Na**	L	115	115	115	0.8	0.6	0.8	0.0	- 0.4	- 1.7	4.6*	5.2*	2.1*^†^	5	6	2.9^†^
	H	158	161	157	0.8	0.5	0.6	- 0.2	- 0.2	- 1.6	3.1*	4.4*	1.4*^†^	4.8	7.5	3.9
**Cl**	L	78	76	75	0.8	0.7	0.8	0.9	4.4	- 3.2	5.1	0.9^†^	2.3^†^	3.8	-0.6^†^	1^†^
	H	118	117	119	1.1	0.6	0.5	0.8	1.3	- 3.3	3.8	6.2	3.5	2.9^†^	4.5	1.5^†^
**Ca**	L	1.48	1.51	1.4	1.6	1.2	1.9	0.1	0.7	- 3.2	10.4*	12.6*	7.5*	3.1	3.6	0.9^†^
	H	3.28	3.35	3.22	0.9	0.9	1.1	0.9	1.6	0.6	7.1*	6.6*	6.8*	4.5	3.8	4.2
*Allowable total error (TEa) calculated by using absolute bias to calculate σ; not marked indicates using percentage bias to calculate σ. ^†^ Sigma values < 3. TEa_CLIA_ - allowable total error derived from US Clinical Laboratory Improvement Amendments 1988 (CLIA ‘88). TEa_WS/T_ - allowable total error derived from the People’s Republic of China Health Industry Standard (WS/T403-2012). Alb - albumin, g/L. ALT - alanine aminotransferase, U/L 37 °C. BT - bilirubin, total, µmol/L. Glc - glucose, mmol/L. CREA - creatinine, µmol/L. Urea, mmol/L. K - potassium, mmol/L. Na - sodium, mmol/L. Cl - chlorides, mmol/L. Ca - calcium, mmol/L.																

For σ_CLIA,_ all 10 assays were above 3σ on the Beckman AU5800 analyser, of which ALT, BT, CREA and Ca achieved 6σ at two levels of QC materials. For the Roche C8000 system, σ_CLIA_ < 1σ for Cl, whereas all other nine items were > 3σ, and BT, CREA, K and Ca achieved 6σ while ALT reached 5σ. For the Siemens Dimension analyser, the σ_CLIA_ value for Glc, Na and Cl was < 3, but Alb, CREA, K and Ca achieved 6σ. Supplementary Figure 2 displays more intuitive σ_CLIA_ levels for the 10 assays at various concentrations using the three analysers. We also calculated σ metrics for the 10 assays based on IQC data from January – June 2017 and observed some differences compared with the σ metrics calculated from the IQC data in June (Supplementary Table 1).

### Comparison of σ Metrics Between Methods

**Figure 1:**
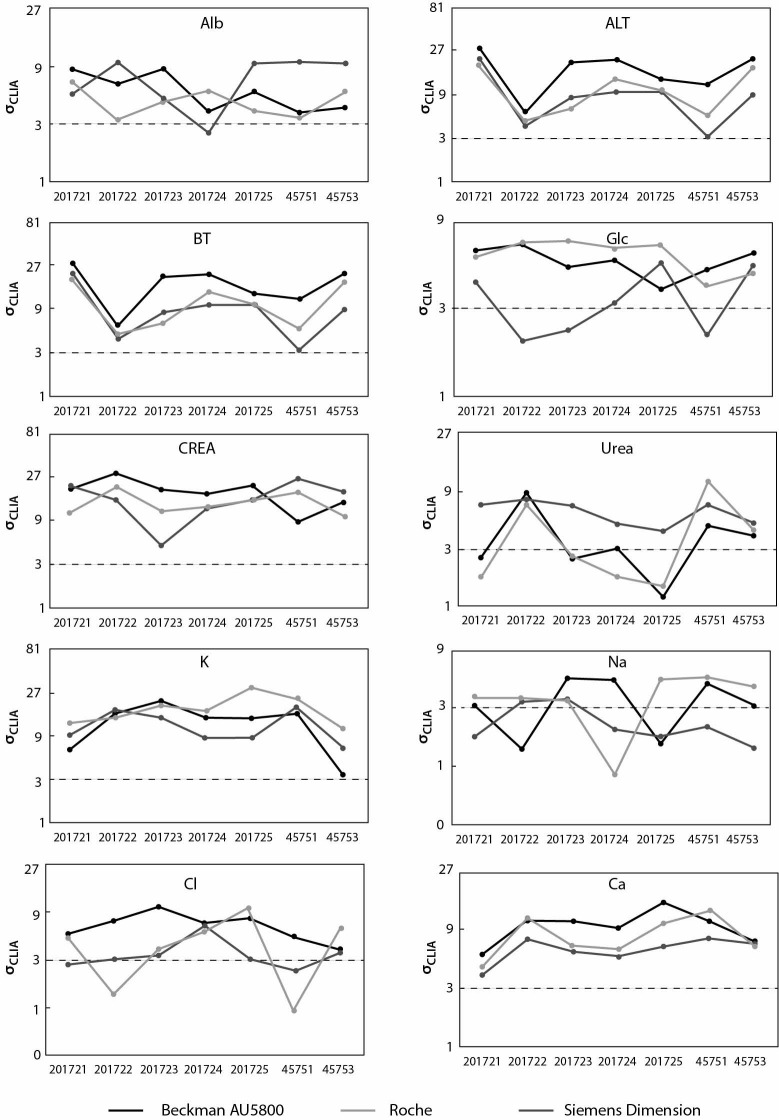
Comparison of σ_CLIA_ values calculated using two methods for the same test item. Note: 201721–201725 represent the lot number of proficiency testing materials, and 45751 and 45752 represent the lot number of Bio-Rad chemistry quality control materials. The horizontal lines indicate the 3σ level line. Alb - albumin, g/L. ALT - alanine aminotransferase, U/L 37 °C. BT - bilirubin, total, µmol/L. Glc - glucose, mmol/L. CREA - creatinine, µmol/L. Urea, mmol/L. K - potassium, mmol/L. Na - sodium, mmol/L. Cl - chlorides, mmol/L. Ca - calcium, mmol/L.

In Figure 1, the differences in σ_CLIA_ as calculated using the two methods and three analysers are shown. For some analytes, the values of σ_CLIA_ derived from the two approaches are significantly different. For example, σ_CLIA_ for the PT-based approach *versus* the IQC-based approach at similar concentrations was 6.5 (201722) *versus* 3.9 (45751) for Alb and 1.4 (201722) *versus* 4.6 (45751) for Na on the Beckman AU5800 analyser. To allow comparison of the differences between the two methods of calculating σ_CLIA_ at similar concentrations, we have listed the σ_CLIA_ values from IQC materials and PT samples in Supplementary Figure 3.

## Discussion

Evaluating the quality of laboratory testing is an important research topic in clinical laboratories. Six Sigma quality standards take bias (system error) and CV (random error) into account to systematically and extensively guide quality management in clinical laboratories while analysing possible causes of error, identifying solutions, better assuring testing quality and optimizing the QC schedule. However, the optimum TEa, bias, CV, and other indicators to calculate 6σ remain unclear, particularly when the sources of bias and CV vary between laboratories. We therefore compared two new approaches to calculate σ metrics as a future reference for the application of 6σ quality management in clinical laboratories.

To our knowledge, the present study is the first to use PT samples to assess imprecision and further calculate σ values. We obtained CV values from these samples to ensure that the same source of bias and CV were used in σ calculations. Given that PT samples typically have five different levels of concentrations, and it is easier to cover different levels of an assay for medical decision-making, this approach conveniently evaluates σ at different concentrations to better indicate analyser performance. A limitation of the PT-based approach is the assessment of short-term imprecision, which may lead to a lower CV and overestimated σ value. According to the manufacturer’s instructions for the PT materials, prepared samples are stable for only seven days, and so are unsuitable for use in long-term evaluations.

To compare differences in σ levels calculated by the two methods, we used the relatively long-term CV calculated in the same month of IQC data in the IQC-based approach, thus accounting for other factors (batch number, instrument status, calibration, personnel, temperature, humidity, *etc.*). Our findings indicate that, for some assays, the σ values derived from the two approaches are significantly different. These differences may clearly have significant outcomes for QC rule selection; for example, a 4σ method requires multi-rule QC while a 6σ method can be controlled by a simple, single-rule QC.

There may be several reasons for the difference between the two approaches. First, there may be obvious differences in analyte concentrations between the IQC control samples and the PT materials. Second, the group mean might not have been appropriate. Third, systematic deviations may have been present, possibly due to the short detection time period when evaluating σ with PT. In addition, the σ value itself is influenced by both bias and imprecision, and in clinical laboratories, multiple factors can influence these parameters. Where the σ value is not satisfactory, the cause should be determined, whether it is a bias or an imprecision issue, and an appropriate solution should be identified. In the PT-based approach, the imprecision was mostly acceptable, and the suboptimal 6σ values may have been mainly attributable to bias. If a laboratory wants to use individualized quality control rules, it may select one method for evaluating σ level and use another method for verification.

We also compared the σ calculation in the IQC-based approach during different time periods and found that the 1- and 6-month values differed significantly for some analytes (Table 4 and Supplementary Table 1). As greater imprecision is expected with prolonged time, 6-month σ values were expected to be lower. However, this was not observed in all cases; for example, Glc (low QC level, 45751) on the Siemens analyser was 2.5 at 1 month *versus* 5.1 at 6 months, and Urea (low QC level, 45751) on the Beckman analyser was 4.7 *versus* 7.2. From the original data analysis, the difference in Glc on the Siemens analyser was due to the larger CV observed in June. The difference in Urea on the Beckman analyser, however, may have been the result of periodic biases with different signs averaged to an ignorable long-term bias. In practice, laboratories must take caution when implementing “Westgard Sigma Rules” in quality control based on σ values, because these values may change continuously with respect to precision and bias arising, for example, from calibrations, reagents, or personnel (*4*). As described previously, σ value between different periods for some analytes may differ, laboratory should monitor the σ level continuously when using individualized quality control rules based on the σ evaluation.

Bias can significantly impact the σ metric. It can (theoretically) be corrected, while imprecision is more difficult to influence. However, bias is generally more difficult to estimate. The most reliable way to do so is to use a reference method. Most studies, including the present study, use group means from EQA data on the same instruments and methods as a target, rather than the reference method. Therefore, the observed bias is only “arbitrary” instead of “true”. External quality assessment peer group evaluation has also been shown to be insufficient in determining analytical quality and may compromise patient care, despite its acceptance by participating laboratories and manufacturers (*14*). This approach is therefore a limitation of the present study, but also represents a common limitation of 6σ for quality management at present, as most routine laboratory testing does not have a reference method that can be conveniently implemented. Determining bias from proficiency testing or global QC reports thus remains the primary approach in current 6σ evaluations. It is therefore critical to select the appropriate group when using group mean to assess bias. In the PT-based approach used in our study, group means were calculated after excluding data more than two standard deviations away from the mean in order to exclude extreme values. Compared with the IQC-based approach, the relatively small number of laboratories using Siemens instruments (< 10) in the PT-based approach may have affected the reliability of the means. This limitation should be considered when selecting an appropriate method for σ calculation.

The TEa is another important parameter in σ calculations, and extensive efforts to understand, establish and unify the quality of testing and analysis are ongoing. In May 2014, the European Federation of Clinical Chemistry and Laboratory Medicine (EFLM) held its first meeting on countermeasures in Milan, Italy, under the theme “Analytical performance targets set 15 years after the Stockholm Conference”. At the conference, experts made in-depth and detailed discussions on the progress and further understanding of setting up analytical performance goals in clinical laboratories in the 15 years after the Stockholm meeting, and also issued a statement of synergies after the meeting (*15*). At present, TEa values are primarily derived from CLIA guidelines, although few reports have used biological variations (*6*, *7*, *9*, *16*, *17*). In China, the Ministry of Health published analytical quality specifications for routine clinical biochemistry (WS/T 403-2012) in 2012, derived from data on within-subject and between-subject biologic variation, while taking into account the quality of analysis currently achievable. However, these standards are expert based and have the objective (at least for CLIA) to set broad quality limits that will include the majority of laboratories; CLIA guidelines, for example, are often considered “loose” in terms of analytical performance. The TEa value selection can lead to significant differences in the evaluation of σ values (*10*). This limitation should be considered when selecting an appropriate TEa for σ calculation.

We also compared the effects of TEa values on σ calculations. The σ_WS/T_ value was significantly lower than that of σ_CLIA_ in most assays, given that TEa_WS/T_ is more stringent than TEa_CLIA_. For some assays, the analyser could not achieve even the 3σ level. In addition, absolute bias was used for the K, Na and Ca assays in the CLIA guidelines, and BT, Glc, Urea and CREA at the low levels, but all percentage bias was used for TEa_WS/T_. Therefore, for low-concentration specimens (201722, 45751), σ_CLIA_ was significantly higher than σ_WS/T_ for BT, CREA, Urea, K and Ca. When screening TEa sources, the source most closely related to the performance for a given laboratory should be selected to ensure continuous improvement in quality management. Laboratories should not pursue the best σ metrics as a laboratory goal, nor should they select the most stringent TEa sources, to avoid unnecessary burden on laboratories.

In the present study, only the reagents for CREA and BT on the Beckman AU5800 system were not obtained from the original manufacturer. The results showed a minimum σ_CLIA_ for BT and CREA of 5.6 and 17.4, respectively, when calculated from proficiency testing samples, and 7.4 and 8.4, respectively, when calculated from IQC. Both results were satisfactory. These findings indicate that both domestic and foreign reagents selected for routine laboratory testing can achieve a high quality level.

The performance of the analysers was also compared. The Beckman AU5800 and Roche C8000 systems each reached 3σ levels for seven assays, while the Siemens Dimension analyser received 3σ levels for five assays. Different assays showed variations in performance among the analysers, although these variations were not significantly different. A laboratory may select an analyser based on assay usage frequency while still considering the σ evaluation, thereby personalizing the selection. Different assays may be also assigned to different instruments based on these results. We found that, for the Siemens Dimension system, σ assessed by both methods had multiple values < 3 (14% for PT samples, 20% for IQC materials). Given that the Siemens Dimension instrument in our laboratory has been in daily use for more than 5 years, it was replaced by a new instrument in December 2017.

In conclusion, both methods of evaluating σ in this study can be used to assess the performance of a specific analyser, despite the observed differences in σ calculated by different methods. In the practical application of σ metrics for QC management, σ should be evaluated multiple times when optimizing a QC schedule.

## References

[1] Forsman RW Why is the laboratory an afterthought for managed care organizations? Clin Chem. 1996;42:813–6. 8653920

[2] Nevalainen D Berte L Kraft C Leigh E Picaso L Morgan T Evaluating laboratory performance on quality indicators with the six sigma scale. Arch Pathol Lab Med. 2000;124:516–9. 10747306 10.5858/2000-124-0516-ELPOQI

[3] Westgard JO Westgard SA Assessing quality on the Sigma scale from proficiency testing and external quality assessment surveys. Clin Chem Lab Med. 2015;53:1531–5. 10.1515/cclm-2014-1241 25719323

[4] Westgard JO Bayat H Westgard SA Planning risk-based SQC schedules for bracketed operation of continuous production analysers. Clin Chem. 2018;64:289–96. 10.1373/clinchem.2017.278291 29097516

[5] Westgard JO Westgard SA Quality control review: implementing a scientifically based quality control system. Ann Clin Biochem. 2016;53:32–50. 10.1177/0004563215597248 26150675

[6] Shaikh MS Moiz B Analytical performance evaluation of a high-volume hematology laboratory utilizing sigma metrics as standard of excellence. Int J Lab Hematol. 2016;38:193–7. 10.1111/ijlh.12468 26847366

[7] Singh B Goswami B Gupta VK Chawla R Mallika V Application of sigma metrics for the assessment of quality assurance in clinical biochemistry laboratory in India: a pilot study. Indian J Clin Biochem. 2011;26:131–5. 10.1007/s12291-010-0083-1 22468038 PMC3107403

[8] Tran MT Hoang K Greaves RF Practical application of biological variation and Sigma metrics quality models to evaluate 20 chemistry analytes on the Beckman Coulter AU680. Clin Biochem. 2016;49:1259–66. 10.1016/j.clinbiochem.2016.08.008 27527571

[9] Nanda SK Ray L Quantitative application of sigma metrics in medical biochemistry. J Clin Diagn Res. 2013;7:2689–91. 10.7860/JCDR/2013/7292.3700 24551613 PMC3919274

[10] Hens K Berth M Armbruster D Westgard S Sigma metrics used to assess analytical quality of clinical chemistry assays: importance of the allowable total error (TEa) target. Clin Chem Lab Med. 2014;52:973–80. 10.1515/cclm-2013-1090 24615486

[11] Clinical and Laboratory Standard Institute (CLSI). User Verification of Precision and Estimation of Bias: Approved Guideline. EP15A3, 3rd ed. Wayne: CLSI, 2014.

[12] CLIA Requirements for Analytical Quality. Available at: https://www.westgard.com/clia.htm/. Accessed February 5th, 2017.

[13] People’s Republic of China Health Industry Standard. (WS/T403-2012), 2012. Available at: http://www.clinet.com.cn/sigmapv/. Accessed November 20th 2017.

[14] Friedecky B Kratochvila J Budina M Why do different EQA schemes have apparently different limits of acceptability? Clin Chem Lab Med. 2011;49:743–5. 10.1515/CCLM.2011.105 21235390

[15] Sandberg S Fraser CG Horvath AR Jansen R Jones G Oosterhuis W Defining analytical performance specifications: consensus statement from the 1st Strategic Conference of the European Federation of Clinical Chemistry and Laboratory Medicine. Clin Chem Lab Med. 2015;53:833–5. 10.1515/cclm-2015-0067 25719329

[16] Huysal K Budak YU Application of sigma metrics for the assessment of quality assurance using the MQ-2000 PT HbA1c analyzer. Biochem Med (Zagreb). 2015;25:416–20. 10.11613/BM.2015.042 26527591 PMC4622199

[17] Ricós C Alvarez V Cava F Garcia-Lario JV Hernandez A Jimenez CV Current databases on biological variation: pros, cons and progress. Scand J Clin Lab Invest. 1999;59:491–500. 10.1080/00365519950185229 10667686

